# Inactivation of TRPM2 Channels by Extracellular Divalent Copper

**DOI:** 10.1371/journal.pone.0112071

**Published:** 2014-11-11

**Authors:** Wenyue Yu, Lin-Hua Jiang, Yang Zheng, Xupang Hu, Jianhong Luo, Wei Yang

**Affiliations:** 1 Key Laboratory of Medical Neurobiology of the Ministry of Health of China, Zhejiang Province Key Laboratory of Neurobiology, Department of Neurobiology, School of Medicine, Zhejiang University, Hangzhou, Zhejiang Province, China; 2 Department of Physiology and Neurobiology, Xinxiang Medical University, Xinxiang, Henan Province, China; 3 School of Biomedical Sciences, Faculty of Biological Sciences, University of Leeds, Leeds, United Kingdom; University of Hull, United Kingdom

## Abstract

Cu^2+^ is an essential metal ion that plays a critical role in the regulation of a number of ion channels and receptors in addition to acting as a cofactor in a variety of enzymes. Here, we showed that human melastatin transient receptor potential 2 (hTRPM2) channel is sensitive to inhibition by extracellular Cu^2+^. Cu^2+^ at concentrations as low as 3 µM inhibited the hTRPM2 channel completely and irreversibly upon washing or using Cu^2+^ chelators, suggesting channel inactivation. The Cu^2+^-induced inactivation was similar when the channels conducted inward or outward currents, indicating the permeating ions had little effect on Cu^2+^-induced inactivation. Furthermore, Cu^2+^ had no effect on singe channel conductance. Alanine substitution by site-directed mutagenesis of His995 in the pore-forming region strongly attenuated Cu^2+^-induced channel inactivation, and mutation of several other pore residues to alanine altered the kinetics of channel inactivation by Cu^2+^. In addition, while introduction of the P1018L mutation is known to result in channel inactivation, exposure to Cu^2+^ accelerated the inactivation of this mutant channel. In contrast with the hTRPM2, the mouse TRPM2 (mTRPM2) channel, which contains glutamine at the position equivalent to His995, was insensitive to Cu^2+^. Replacement of His995 with glutamine in the hTRPM2 conferred loss of Cu^2+^-induced channel inactivation. Taken together, these results suggest that Cu^2+^ inactivates the hTRPM2 channel by interacting with the outer pore region. Our results also indicate that the amino acid residue difference in this region gives rise to species-dependent effect by Cu^2+^ on the human and mouse TRPM2 channels.

## Introduction

The TRPM2 channel belongs to the melastatin subfamily of the mammalian transient receptor potential (TRP) channels, which share several conserved domains with other TRPM channels, such as the TRPM homology domains (MHD domains) in the N-terminus and the TRP box and coiled-coil domain in the C-terminus [1]–[4]. The TRPM2 channel is a homo-tetramer and each subunit contains six transmembrane segments with a pore-forming region between the fifth and sixth segments and intracellular N- and C-termini [5]. The TRPM2 channel is a non-selective cation channel and permeates calcium ion, and is activated by intracellular ADP-ribose (ADPR) [1], [6] or intracellular calcium [7]–[10]. Accumulating evidence indicates that the TRPM2 channel plays an important role in a number of physiological and pathophysiological processes, including neurodegeneration, immunological functions, insulin release [11]–[15]. Previous studies showed that the TRPM2 channel can undergo rapid inactivation upon exposure to extracellular proton and Zn^2+^ that interact selective residues in the pore region [9], [16], [17]. Mutation of the residues in the pore region can strongly alter the channel inactivation. Thus, the disease-associated P1018L mutation conferred rapid inactivation of the hTRPM2 channel, whereas manipulation of the pore region by site-directed mutagenesis resulted in a TRPM2-LDE mutant channel that exhibited no inactivation, suggesting alterations in the conformation and structure of the pore region represent an important molecular mechanisms of the TRPM2 channel inactivation [18], [19].

Cu^2+^ is the third abundant trace metal in the human body, and plays a critical role in a variety of physiological and pathological conditions. Cu^2+^ is a cofactor for a variety of enzymes, and relates to the formation of reactive oxygen species. Like zinc, excessive Cu^2+^ is toxic for neurons [20], [21]. Cu^2+^ is involved in several human diseases [22]–[24], and the Cu^2+^ chelators have been intensively used as therapeutic treatments Cu^2+^ related diseases, such as Wilson's disease and cancer [25]. Several studies suggest Cu^2+^ and Zn^2+^ regulate cell functions via altering the activity of a variety of ion channels [26], [27]. For example, Cu^2+^ reduces the tonic inhibition of neurons by blocking the GABA_A_ receptors [28]. Therefore, elucidating the mechanisms regulating ion channels by Cu^2+^ is critical for a better understanding of its physiological and pathological roles in humans.

It is well known that Cu^2+^ can activate, modulate or inhibit ion channels. For example, Cu^2+^ activates the TRPV1 and TRPA1 channels [29], [30] and, by contrast, Cu^2+^ inhibits endothelial Na^+^ channels [31], BK and Shaker K^+^ channels [32]. A recent study has reported that extracellular Cu^2+^ induces the hTRPM2 channel inactivation [33], but the underlying molecular or structural basis still remains unclear. Here, using site-directed mutagenesis and patch-clamp recording, we identified His995 in the pore region to be crucial in Cu^2+^-induced hTRPM2 channel inactivation. In addition, the mTRPM2 channel is insensitive to Cu^2+^ and such a species-dependent effect by extracellular Cu^2+^ arises from replacement of His995 in the hTRPM2 channel with glutamine at the equivalent position in the mTRPM2 channel.

## Materials and Methods

### Clones, cells and molecular biology

The cDNAs encoding the hTRPM2 and mTRPM2 were kindly provided by Dr AM Scharenberg (Washington University, USA) and Dr Y Mori (Kyoto University, Japan), respectively. Tetracycline-inducible HEK293 cells stably expressing hTRPM2 was kindly provided by Dr AM Scharenberg. The point mutations were introduced by site-directed mutagenesis [34] and confirmed by sequencing. Human embryonic kidney (HEK) 293 cells were used to transiently express wild-type (WT) and mutant channels. HEK293 cells were maintained in DMEM/F-12 medium supplemented with 50 units/ml penicillin, 50 µg/ml streptomycin (Gibco, USA) and 10% fetal bovine serum (Gibco, USA). Cell transfection was described previously [4]. Tetracycline-inducible hTRPM2-expressing HEK293 cells were used in some experiments and the expression of the hTRPM2 channel was induced by adding 1 µg/ml tetracycline in the culture medium for 12–24 h before use.

### Electrophysiology

Whole-cell current recordings were performed using an Axonpatch 200B amplifier at room temperature as described previously [4]. The extracellular solution contained (in mM): 147 NaCl, 2 KCl, 1 MgCl_2_, 2 CaCl_2_, 10 HEPES, and 13 glucose, pH 7.4. The intracellular solution contained (in mM): 147 NaCl, 0.05 EGTA, 1 MgCl_2_, 10 HEPES, and 0.5 ADPR, pH 7.3. Thus, the currents were mainly carried by Na^+^. The cell membrane potential was held at 0 mV. To record ADPR-induced currents, voltage ramps with 500 ms duration from −100 mV to 100 mV were applied every 5 s. The glass microelectrodes with resistance of 3–5 MΩ were used. Single channel activity recordings were performed using a HEKA EPC10 amplifier controlled with PatchMaster software (HEKA), and carried out in the outside-out configuration as previous described [17]. Data were acquired at 10 kHz and filtered offline at 50 Hz. CuSO_4_ stock solution was prepared by dissolving in the extracellular solutions. Changes of extracellular solutions and applications of Cu^2+^, clotrimazole (CLT), 2-ME and EDTA were achieved using an RSC-160 system (Biologic Science Instruments) with a solution change time of ∼300 ms [4].

### Data analysis

The data are presented in the text and figures, where appropriate, as mean ± S.E. values. The inhibition was expressed by the currents in the indicated solutions as the percentage of the currents before the solution changes, and the kinetics were estimated by determining the time required for 90% inhibition (t_90%_). Single-channel conductance was estimated from all-point histograms constructed from the current events recorded at −80 mV. A double-Gaussian function was fitted to the histograms as previously described [35]. Statistical analysis was performed using Student's t-test with p<0.05 designated as significant difference.

## Results

### Extracellular Cu^2+^ induces hTRPM2 channel inactivation

**Figure 1:**
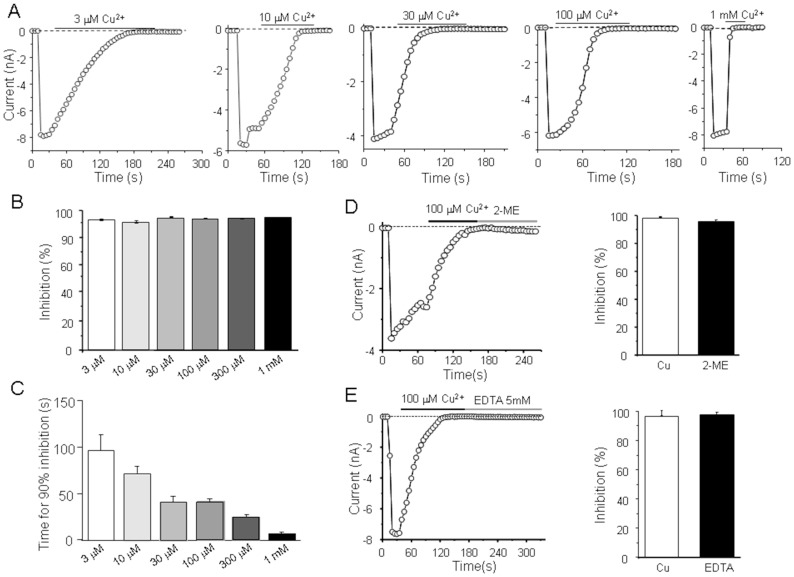
TRPM2 open channels inactivated by extracellular Cu^2+^. (A) Representative recordings of the inward currents evoked by 500 µM ADPR at −80 mV, using a 500 ms voltage ramp of −100 mV to +100 mV applied every 5 s, before and after exposure to the indicated Cu^2+^ concentrations. The dotted lines indicate zero currents. (B–C) Summary of the percentage inhibition (B) and time required for inward current amplitude reached 90% inhibition after Cu^2+^ exposure (C). (D) Left panel, the ADPR-induced inward currents when fully inhibited by 100 µM Cu^2+^ were not reversed after treating with 20 µM 2-ME; Right panel, summary of the current recovery during exposure to 2-ME. (E) Left panel, the ADPR-induced inward currents when fully inhibited by 100 µM Cu^2+^ were not reversed after treating with 5 mM EDTA; Right panel, summary of the current recovery during exposure to EDTA. Residual current expressed as the percentage of the currents immediately before exposure to Cu^2+^ is 3.3±1.7% after inactivation by Cu^2+^, which returned to 2.6±0.9% after washing with EDTA. In 2-ME group, residual current changed from 1.8±0.5% to 2.0±0.7%. The number of cells examined in each case is 4–6.

We first investigated the effect of extracellular Cu^2+^ on the open hTRPM2 channels. The activity of the hTRPM2 channels was induced by application of ADPR (500 µM) in the pipette solution, as illustrated by the inward currents at −80 mV (Fig. 1A). Perfusion with 3 µM Cu^2+^ abolished the hTRPM2 channel currents in several minutes, as reported by a recent study [33]. While the hTRPM2 channels were completely inhibited by Cu^2+^ independently of concentrations from 3 µM to 1 mM, the time required for 90% inhibition (t_90%_) was concentration-dependent from 98.4±16.7 s (n = 4) at 3 µM to 7.1±1.5 s (n = 5) at 1 mM (Fig. 1B). The inhibitory effect of Cu^2+^ was irreversible on washout, which suggests that Cu^2+^ induced channel inactivation like proton and zinc [16], [17], [36]. Such inactivation could result from either channel inactivation or tight binding of Cu^2+^ to the hTRPM2 channels. To clarify this issue, we applied 2-ME or EDTA to chelate Cu^2+^, after Cu^2+^ induced complete inhibition of ADP-induced currents. The inhibition was still not rescued by treating with the 2-ME or EDTA for up to two min (Fig. 1C, D), which suggests that Cu^2+^ may not bind with the TRPM2 after TRPM2 inactivation, or alternatively Cu^2+^ tightly binds with the hTRPM2 channels in the small pocket and both EDTA and 2-ME cannot access.

**Figure 2:**
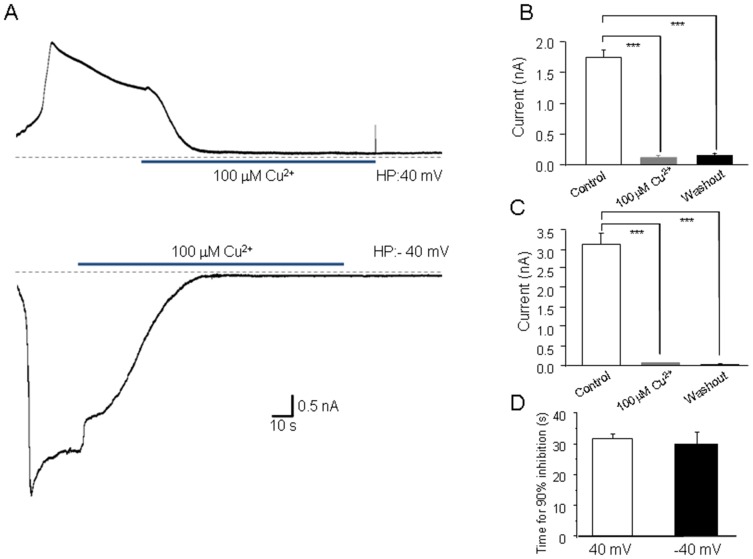
Voltage-independent effects by extracellular Cu^2+^ on inward and outward TRPM2 channel currents. (A) ADPR-induced currents mediated by the hTRPM2 channels at a holding membrane potentials (HP) of +40 mV (outward currents) or −40 mV (inward currents) and the effect of 100 µM Cu^2+^. The dotted lines indicate the baseline. (B–C) Summary of the outward or inward current amplitude before and after exposure to Cu^2+^ and upon washout, as shown in (A). The number of cells examined in each case is 4. ***, p<0.005 compared with the currents before and after exposure to the indicated Cu^2+^. (D) Summary of the time for 90% inhibition at both 40 mV and −40 mV, there is no significant difference between these groups.

**Figure 3:**
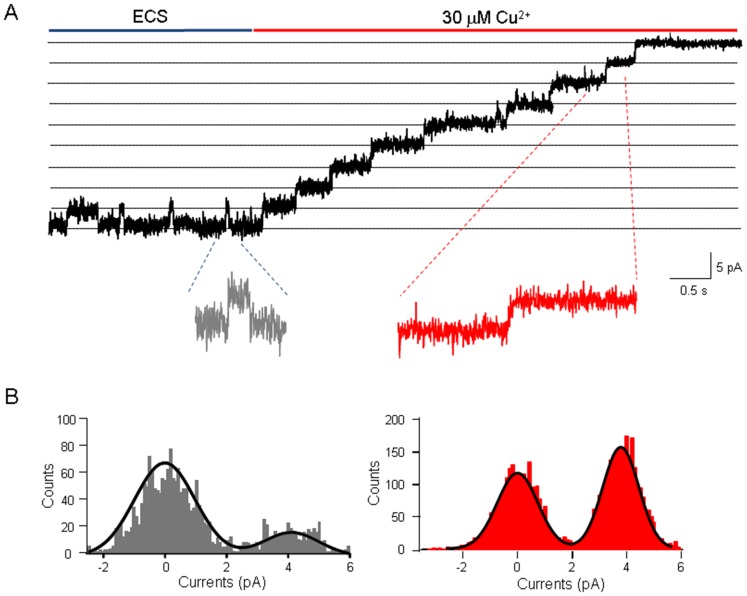
Effects of external Cu^2+^ on human TRPM2 single channel conductance. (A) Representative recordings in the outside-out configuration of the effects of 30 µM Cu^2+^ on ADPR-induced TRPM2 currents (in red). Single channel events are clearly visible in the expanded traces illustrated below. (B) The grey and red histograms of single channel events indicated the exposure in ECS and 30 µM Cu^2+^, respectively. The superimposed curve represents a fit of a double­Gaussian function.

Our previous study showed that Zn^2+^ inactivates the hTRPM2 channel and such inactivation is strongly affected by the permeant ions [17]. It was thus interesting to know whether Cu^2+^-induced hTRPM2 inactivation was similar as that by Zn^2+^. Unlike Zn^2+^, 100 µM Cu^2+^ still inhibited irreversibly the hTRPM2 channel currents at 40 mV (Fig. 2A), which suggests the hTRPM2 inactivation induced by Cu^2+^ may be different from that by Zn^2+^. Moreover, we investigated whether Cu^2+^ affects the single channel conductance using the patch-clamp recording in outside-out configuration. As shown in Fig. 3, the single channel conductance of the hTRPM2 channels was not changed by Cu^2+^.

### His995 is a key residue in the outer pore in Cu^2+^-induced inactivation

**Figure 4:**
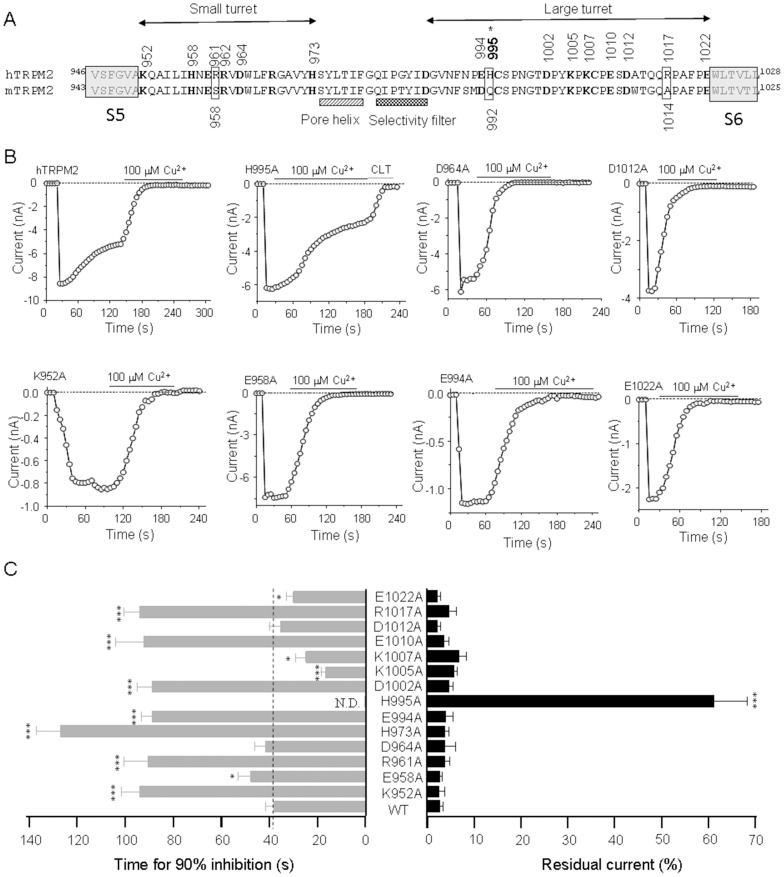
Alanine substitution of Cu^2+^-binding candidate residues in the outer pore of hTRPM2 channel. (A) The amino acid sequences of the pore region between the S5 and S6 of the hTRPM2 channel; the residues examined in this study are numbered and highlighted in bold. Residues in the extracellular ends of S5 and S6 are indicated in the left and right shading boxes, respectively. Alanine substitutions leading to loss of function are indicated by asterisks. (B) Representative recordings of the ADPR-induced currents in HEK cells expressing WT or the indicated mutant channel before and after exposure to 100 µM Cu^2+^ (denoted by the black bars). The currents for the H995A mutant channels show incomplete inhibition by Cu^2+^ and complete inhibition by subsequent application of 20 µM CLT (denoted by the grey bars). The dotted lines indicate zero currents. (C) Summary of the time for 90% inhibition (left) and the residual currents upon exposure to Cu^2+^ (right). The dotted lines indicate the time or residual currents for the WT channel. The number of cells examined in each case is 3–22. The mutant channels showing significant difference from the WT channels are indicated in parentheses, *, p<0.05, **, p<0.01, ***, p<0.005.

Metal ions can interact with polar amino acid like histidine, glutamate, aspartate, and lysine [9], [16], [36], [37]. Several studies have identified histidine, cysteine, aspartic acid, arginine and glutamine determine Cu^2+^-induced channel inhibition or inactivation, such as the P2X7 receptor [38], GABA_A_ receptor [28], ENaC channel [31], BK channel [32]. In addition, a previous study of olfactory CNG channel gating suggests that the extracellular pore region is importantly involved in the channel gating [39]. Our previous studies have identified several amino acids which are responsible for channel inactivation induced by proton and Zn^2+^ (Fig. 4A). We hypothesized that Cu^2+^ may interact with similar amino acid residues in the pore region which Zn^2+^ binds to (Fig. 3). We introduced alanine substitution into the candidate amino acid residues as described in previous studies [16], [36], and determined the effects of 100 µM Cu^2+^ on the functional mutant channels. All of these mutants did not strongly resulted in strong channel inactivation as illustrated by several examples in Fig. 4. Representative current traces of the hTRPM2 mutant channels are illustrated in Fig. 4A, and the mutational effects on the Cu^2+^-induced inhibition and inactivation are summarized in Fig. 4B. Several mutants, albeit still be inactivated by Cu^2+^, exhibited significantly slower inactivation kinetics, such as K952A (88.8±6.7 s), R961A (92.3±11.7 s), H973A (126.9±10.3 s), D994A (88.7±6.1 s), D1002A (88.8±4.6 s), E1010A (90.6±9.8 s) and R1017A (90.4±7.8 s) as compared to the WT channel (48.5±2.5 s) (Fig. 4A, B). On the contrary, some other mutations accelerated inactivation kinetics, including K1005A (16.7±1.9 s), K1007A (32.9±7.6 s), E1022A (38.2±3.4 s) (Fig. 4B). Strikingly, the H995A mutant channel was insensitive to inactivation by 100 µM Cu^2+^ (Fig. 4A, B). These results suggest that Cu^2+^ inactivates the hTRPM2 channel via engaging the outer pore region.

### Cu^2+^ accelerates the inactivation of hTRPM2 P1018L mutant channel

**Figure 5:**
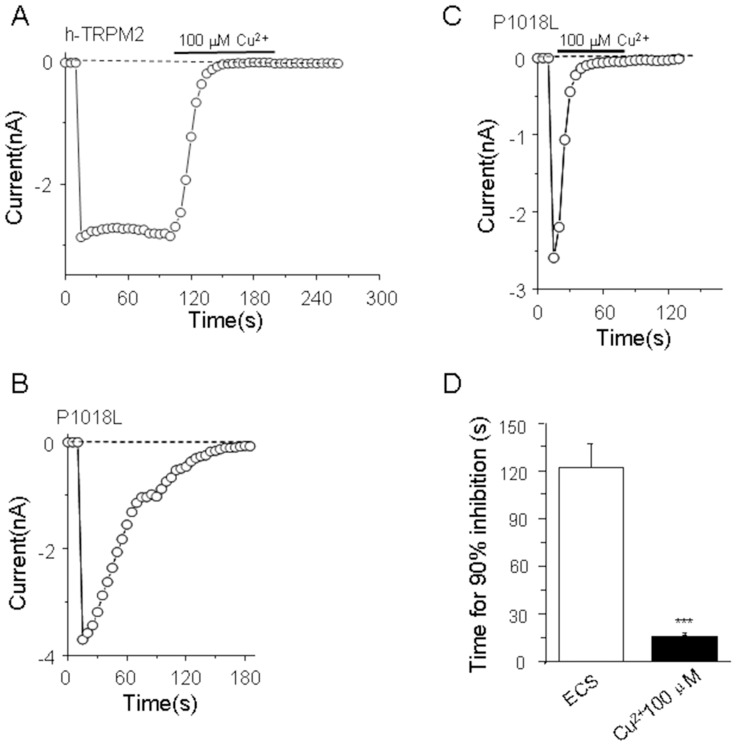
P1018L mutation facilitates Cu^2+^-induced hTRPM2 channel inactivation. (A) The inward current of cell transfected with human TRPM2 is blocked by Cu^2+^. (B) P1018L mutant exhibited strong channel inactivation in extracellular solution (ECS). (C) Cu^2+^ accelerated the P1018L mutant channel inactivation. (D) Summary of the time for 90% inhibition after exposure to Cu^2+^. The number of cells examined in each case is 3–13. The significant difference is indicated in parentheses, ***, p<0.005.

A previous study showed that the P1018L mutation in hTRPM2 resulted in channel inactivation [18], it is interesting to know the effect of extracellular Cu^2+^ on this mutant channel. Consistent with the previous study [18], the hTRPM2 P1018L mutant channel exhibited strong channel inactivation (121.6±14.8 s) (Fig. 5A, B). Interestingly, 100 µM Cu^2+^ significantly accelerated the inactivation kinetics of the hTRPM2 P1018L mutant chanel (15.3±0.7 s) (Fig. 5C, D).

### Mouse TRPM2 channel shows insensitivity to Cu^2+^-induced inactivation

**Figure 6:**
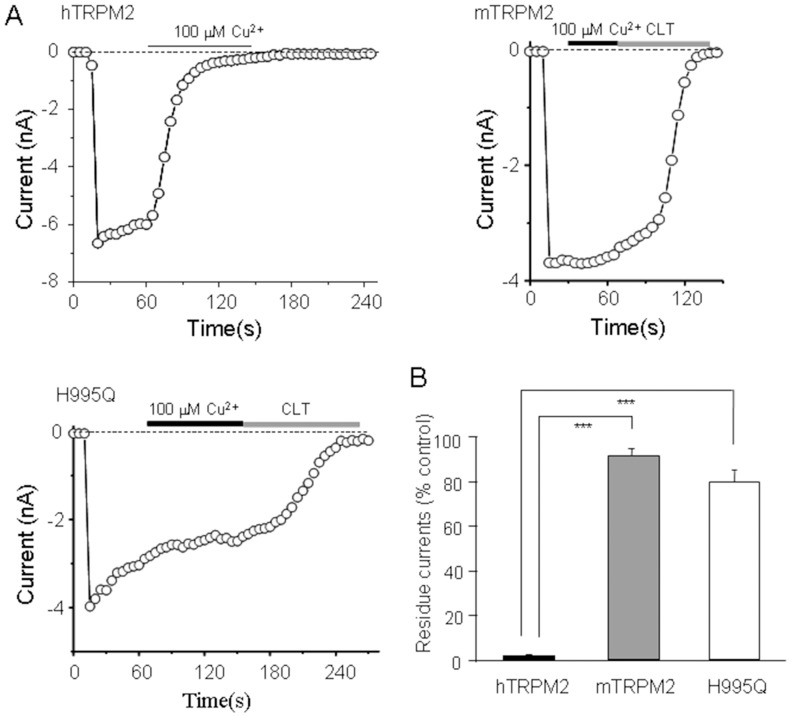
His^995^ is essential in Cu^2+^ interacting with human TRPM2 channel. (A) Recordings of the inward currents in cells expressing the human, mouse wild-type or mutant TRPM2 channels. (B) Summary of the effects of Cu^2+^ on these three channels. The number of cells examined in each case is 3–5. The significant difference is indicated in parentheses. ***, p<0.005.

Species difference exists between the human and mouse TRPM2 channels in terms of inactivation of the TRPM2 channels by protons and zinc, as a result of difference in the amino acid sequences of the pore region [17], [36]. By comparing with the sequences, three residues in the pore region of the hTRPM2 channel are different with those of the mTRPM2. Arg961, His995 and Arg1017 residues in the hTRPM2 are replaced with Ser958, Gln992, and Ala1014 in the mTRPM2, respectively (Fig. 4A). The results that His995 plays a crucial role in Cu^2+^-induced hTRPM2 channel inactivation suggest that Cu^2+^ may not strongly interact with the pore region of the mTRPM2 channel to induce inactivation of the mTRPM2 channel. To address this hypothesis, we expressed the mTRPM2 channels in HEK293 cells and determined the effect of Cu^2+^ on the mTRPM2 channels. As shown in Fig. 6A, 100 µM Cu^2+^ failed to induce significant inhibition/inactivation of the mTRPM2 channels. We further generated the construct expressing the H995Q hTRPM2 mutant channel. The H995Q mutation almost completely abolished the hTRPM2 channel inactivation induced by Cu^2+^, even better than the H995A mutation (Fig. 6B). These results provide further evidence to indicate that His995 is critical in determining hTRPM2 channel inactivation by Cu^2+^.

## Discussion

In this study, we found that extracellular Cu^2+^ inactivates the human but not the mouse TRPM2 channel, and revealed a striking species-dependent effect. Moreover, we identified several residues are involved in this process, particularly His995 which is the key residue in determining the hTRPM2 channel inactivation induced by Cu^2+^.

### Cu^2+^-induced TRPM2 inactivation is independent on electrostatic repulsion and channel inhibition

Unlike the actions of extracellular Cu^2+^ on other ion channels, the present study shows Cu^2+^ induced an irreversible and concentration-independent inhibition of the hTRPM2 channel. Moreover, we found that Cu^2+^ induced similar TRPM2 inactivation at the holding potential of −40 mV driving inward currents, as at 40 mV, driving outward currents. This is different with the membrane potential-dependent action of zinc shown in our previous study [17]. Thus, the action of Cu^2+^ may mechanistically differ from the action of zinc [17]. Remarkably, our data indicate that Cu^2+^-induced hTRPM2 inactivation is independent on electrostatic repulsion, suggesting that Cu^2+^ binds with the residues outside the electrical field and induces conformational changes that lead to hTRPM2 channel inactivation. Both EDTA and 2-ME failed to reverse the Cu^2+^-induced channel inactivation. One simple explanation is that Cu^2+^ induces substantial conformational changes that prevent accessibility of such Cu^2+^ chelators.

### His995 is the key residue determining Cu^2+^-induced inactivation of human TRPM2 channel

As we mentioned before, many studies have identified several residues interact with Cu^2+^, such as histidine, cysteine, glutamate, aspartic acid and arginine. By alanine scanning, we have shown several residues in the pore region that are involved in the Cu^2+^-induced inactivation of the hTRPM2 channel (Fig. 4). Particularly, the H995A mutation that is located at the large turret of the pore region strongly attenuated the hTRPM2 channel inactivation by Cu^2+^ (Fig. 4). Although several other residues replaced by alanine did not prevent the hTRPM2 channel inactivation by Cu^2+^, the kinetics were substantially changed at these mutant channels (Fig. 4). Specifically, K952A, R961A, H973A, D994A, D1002A, E1010A and R1017A mutations slowed down the inactivation kinetics (Fig. 4A, B), whereas K1005A, K1007A and E1022A resulted in faster inactivation kinetics (Fig. 4B). These findings support that Cu^2+^ induces the hTRPM2 channel inactivation by acting on the outer pore region. In future, it is important to uncover the conformational changes that give rise to Cu^2+^-induced hTRPM2 channel inactivation.

### Species specific effects of Cu^2+^ on human and mouse TRPM2 channels

The TRPM2 channels are highly conserved in different species including the human and mouse. Our previous studies found three different residues in the pore region, Arg^961^/Ser^958^, His^995^/Gln^992^, Arg^1017^/Ala^1014^, between the human and mouse TRPM2 channels and showed that His^995^/Gln^992^ is important in determining the different kinetics of channel inactivation induced by proton or Zn^2+^ between the human and mouse TRPM2 channels [17], [36]. Here, we have shown that all of these three sites are critical for the hTRPM2 channel inactivation induced by Cu^2+^, which is confirmed by the observation that there was no mTRPM2 channel inactivation by Cu^2+^ (Fig. 6). Taken together, the previous and present studies suggest that the hTRPM2 channel is more sensitive to Cu^2+^, Zn^2+^ and proton than the mTRPM2 channel. Many lines of evidence have indicated that Cu^2+^ is involved in the pathogenesis of neurodegenerative disorders such as Alzheimer's disease, Parkinson's disease and prion disease [40], [41]. On the other hand, accumulating evidence implicates that the TRPM2 channels as an oxidative stress sensor is also related to these mental disorders [5]. In future, it is interesting to investigate whether the role of Cu^2+^ in the neurodegenerative disorders is related to the hTRPM2 channel. However, according to our results, the striking species difference between the human and mouse TRPM2 channels suggest unsuitable to use mice as animal models to investigate the regulation of TRPM2 channels by Cu^2+^ in relation to the human diseases.

### Comparing the effects on TRPM2 channels induced by Cu^2+^ and Zn^2+^

Both Zn^2+^ and Cu^2+^ are metal ions, but the Cu^2+^-induced TRPM2 channel inactivation is different with that by Zn^2+^. Firstly, the key residues that determine the TRPM2 inactivation by Cu^2+^ and Zn^2+^ are different. Secondly, Cu^2+^-induced TRPM2 channel inactivation is independent on the membrane potential and/or the direction of Na^+^ permeation (Fig. 2). Finally, the Cu^2+^-induced inactivation was accelerated by the K1005A, K1007A, E1022A mutations, however, the Zn^2+^-induced inactivation was accelerated by the D964A, H973A, K1005A, K1007A, R1017A, and E1022A mutations. The Cu^2+^-induced inactivation was slowed by the K952A, R961A, H973A, D994A, D1002A, E1010A and R1017A, whereas the Zn^2+^-induced inactivation was slowed by H958A, E994A, H995A, E1010A, and D1012A. Although there are some mutations present the similar effect on the TRPM2 channel inactivation by Cu^2+^ and Zn^2+^, many mutations resulted in the opposite effects between Cu^2+^-induced inactivation and Zn^2+^-induced inactivation. These differences suggest the critical site of Cu^2+^-binding overlap with but differ from the Zn^2+^-binding site and some differences in the molecular mechanisms in the TRPM2 inactivation induced by Cu^2+^ and Zn^2+^. However, there was no effect of Cu^2+^ on the single channel conductance as previously reported for Zn^2+^ [33], which suggests that both Cu^2+^ and Zn^2+^ induce TRPM2 inactivation without affecting the ion conducting pathway. Future study is required to know how the TRPM2 channels undergo conformational changes during the inactivation during Cu^2+^ or Zn^2+^ treatment.

Our data have shown that the P1018L mutation located at the pore region of hTRPM2 resulted in strong channel inactivation (Fig. 5) as reported previously [18]. We investigated whether hTRPM2 channel inactivation induced by Cu^2+^ and P1018L mutation occurred through similar mechanisms. Our result showed that Cu^2+^ significantly accelerated the inactivation kinetics of the hTRPM2 P1018L mutant channel (Fig. 5), indicating that Cu^2+^ can induce strong channel inactivation of this mutant channel. Taken together, our results suggest that TRPM2 inactivation is complex, it will be interesting to know how to induce channel inactivation by these different factors in the future.

In summary, we have shown that extracellular Cu^2+^ inactivates the hTRPM2 channel independently of the ion conducting pathway. By introducing point mutation, we identified multiple residues, especially His995, in the outer pore region that are involved in Cu^2+^-induced hTRPM2 channel inactivation. We also discovered that the mTRPM2 channel is insensitive to Cu^2+^. These findings open a new way for us to understand the roles of TRPM2 channel in Cu^2+^ related physiological and pathological processes.
